# Acute phase protein concentration in pharyngeal swabs from clinically healthy commercial dairy calves

**DOI:** 10.1186/s13028-023-00714-w

**Published:** 2023-11-25

**Authors:** Mette Bisgaard Petersen, Nynne Capion

**Affiliations:** Department of Veterinary Clinical Sciences, Faculty of Health and Medical Sciences, University of Copenhagen, Copenhagen, Denmark

**Keywords:** Acute phase proteins, Dairy calves, Saliva, Serum, Tracheal aspirate

## Abstract

**Background:**

Early diagnosis of disease in calves is crucial for fast recovery and prudent use of antibiotics. The serum concentration of acute phase proteins (APPs) is up- or downregulated in response to tissue injury and has been studied widely in human medicine. There is growing interest in using APPs as biomarkers for different diseases and as a tool to initiate and monitor treatment in veterinary medicine as well. The concentration of APPs in saliva in healthy calves has not been established and the use of pharyngeal swabs offers a non-invasive alternative to blood sampling. Pharyngeal swabs, tracheal aspirate (TA) and blood samples were collected from 84 clinically healthy commercial dairy calves and analyzed for the APPs serum amyloid A (SAA), haptoglobin (Hp) and lipopolysaccharide binding protein (LBP).

**Results:**

We found detectable concentrations of SAA, Hp and LBP in pharyngeal swabs from calves, as well as in TA and serum. There were no biologically interesting correlations between the SAA concentrations in serum and TA or pharyngeal swabs. This also applied to Hp and LBP concentrations in serum and TA or pharyngeal swabs.

**Conclusions:**

SAA, Hp and LBP can be measured in saliva and TA from calves, but there was no correlation between the specific APP concentration in serum and pharyngeal swab or TA. There was a considerable technical variation in the sampling method for both pharyngeal swab and TA, and further validation of the methods is needed.

## Background

An acute phase protein (APP) is a protein in blood and other body fluids, that plays a role in the systemic acute phase response to a local infection or tissue injury. APPs are unspecific, meaning there are many different proteins that respond differently as a systemic response to a local or systemic inflammation. The concentration of APPs may either increase or decrease in blood and other fluids. However, the response does not differentiate between the agent responsible for the inflammation or type of tissue injury [1]. APPs have been studied widely in human medicine as biomarkers for a variety of diseases and as a tool to initiate and monitor treatment. In veterinary medicine there is growing interest in using APPs with the same purpose.

Serum amyloid A (SAA), haptoglobin (Hp) and lipopolysaccharide binding protein (LBP) have been correlated to different diseases and disease states in cows and calves [1]. Reference values for some serum APPs in calves have also been established to distinguish between healthy and diseased animals [2–4].

SAA, Hp and LBP have different characteristics and are expressed in different quantities in different body fluids and different health states [5–7]. There are differences in the speed at which the concentration of APP rises. Serum LBP and SAA seem to rise earlier in the disease course than Hp [8, 9]. Prohl et al. [10] found that LBP and Hp, amongst other APPs, can be measured in bronchoalveolar fluid (BALF) in both healthy calves and experimentally infected calves with respiratory disease. LBP changes in BALF were both associated with changes in respiratory score and systemic LBP response, and LBP seems to be a very promising candidate for a local marker of respiratory disease in calves [10]. The concentration of Hp and SAA in BALF in calves with bronchopneumonia were higher compared to clinically healthy calves and the intensity in the increase of APP was higher in BALF than serum [11]. These findings indicate that the detection of APPs in BALF can be used to detect respiratory disease in calves.

An easier and less invasive method of detecting APPs from the respiratory tract would be to measure APPs in saliva, e.g., in pharyngeal fluid. In pigs, Hp and C-reactive protein (CRP) have been measured in saliva, and the concentration of Hp in saliva mimicked the concentration in serum, just at a lower concentration [12, 13]. In addition, concentrations of Hp and CRP in saliva were able to differentiate between healthy and diseased pigs and APP concentrations in saliva could be considered as an alternative to serum for detection of unspecific disease in pigs [13]. In cows, it is possible to measure SAA in saliva and there is a positive, but weak, correlation between subclinical mastitis and salivary SAA [14]. SAA, Hp and LBP can be measured in saliva in calves [15], however the authors do not explain the sampling method or the age of the calves. With the overall purpose of establishing a calf side test for the detection of respiratory inflammation, including an assessment of treatment effects and evaluation of prognosis, concentrations of APPs in the bovine pharynx needs to be explored.

The aim of this study was therefore to investigate if SAA, Hp and LBP can be measured in saliva from pharyngeal swabs and tracheal aspirate (TA) from healthy calves.

## Methods

### Animals and sample collection

The study protocol was approved by the Danish Animal Experiments Inspectorate (Approval number, 2021-15-0201-01099). Samples were collected during autumn of 2018 and winter of 2019. Eighty-four healthy commercial Holstein dairy calves aged 2–29 days from nine Danish dairy herds were enrolled in the study. The herds were convenience sampled as part of a large study about calf health. Records of previous disease and treatment was not available, but none of the calves were dehorned prior to sampling. All single housed calves were taken into consideration, and both bulls and heifers were included. All enrolled calves had to be clinically healthy and to determine the health-status, each calf undervent a clinical examination (CE) and thoracic ultrasonography at the day of the visit. Based on the CE, calves were only included if they were bright, alert and responsive, and did not show any of the following clinical signs: nasal and/or ocular discharge, a rectal temperature above 39.0 °C, abnormal breathing or abnormal lung sounds during auscultation and were given a thoracic ultrasonography score (TUS) of 0 or 1 as described by Ollivett and Buczinski [16]. Thoracic ultrasonography was performed on non-sedated calves, the hair coat stayed untouched and ethanol was used as contact medium. The entire lung field was scanned from the 12th intercostal space cranial to the first intercostal space on both sides.

After CE and TUS, the calves were lightly sedated with Xylazine (0.06 mg/kg) intravenous. A non-guided TA sample was collected with a calf flush catheter (proVETnordic, Ref.: 20,096). The catheter was placed in the trachea until resistance. Fifty mL isotonic NaCl was flushed into the lungs and as much as possible was aspirated. Two mL were transferred to a 2 mL EDTA tube and the rest was transferred to a 50 mL plain tube (Sarstedt AG & Co. KG, Germany). It was possible to aspirate 22–38 mL, with an average of 32 mL. Thereafter, four long cotton swabs were placed as far in pharynx as possible and rotated until soaked in saliva. The cotton swabs were placed in cryotubes containing 1 mL phosphate buffer saline (PBS). Finally, blood was drawn from the jugular vein into three serum tubes (8.5 mL, SST^TM^II *Advance).*

### Preparation of samples

The serum tubes were kept at room temperature for at least 2 h, centrifuged at 4327 Rpm for 10 min and the supernatant were distributed into cryotubes, which were kept at – 80 °C until further analysis.

The pharyngeal swabs were stored in a PBS solution for 24 h at room temperature. Then the cotton swabs were removed, and the PBS liquid was stored at – 80 °C until further analysis. A cotton swab can contain approximately 100–110 µL of saliva and concentrations of acute phase proteins are therefore multiplied by 11, as the cotton swab was stored in 1 mL of PBS.

The TA liquid was kept at 5 °C for maximum 2 days, then it was centrifuged at 1410 Rpm for 10 min at 4 °C and the supernatant were kept at – 80 °C until further analysis.

### Laboratory analysis

Serum, pharyngeal swabs, and TA samples were analysed for SAA, Hp and LBP. All analyses were performed at the Veterinary Diagnostic Laboratory, Frederiksberg, Denmark. The SAA were analysed with multispecies SAA ELISA (Tridelta Development Ltd., Maynooth, County Kildare, Ireland). The Hp with a colorimetric assay (phase, haptoglobin assay, Tridelta Development Ltd., Maynooth, County Kildare, Ireland). Calibration was performed using a calibrator set from the same manufacturer using ADVIA 1800 (Siemens Healthineers, Ballerup, Denmark). Analysis for LBP were done with the ELISA kit HK503 (Hycult Biotech, Uden, The Netherlands). The serum samples were run according to the manufacturer´s manual. None of the used test kits are validated for use with either saliva or TA. The pharyngeal swabs and TA samples were therefore run according to the manufacturer´s manual for serum, with the only difference being that they were run undiluted. This was done because both sample types were already diluted by the sampling method, and due to concerns about not getting a readable signal.

### Statistical analysis

All data management and analyses were done in R version 3.2.2 [17]. When modelling the correlation between serum LBP and age, serum LBP concentration was log-transformed to improve the normality of the residuals. The goodness-of-fit of the model was estimated using r^2^. The correlation between the APP concentration in serum and pharyngeal swabs, serum and TA and, pharyngeal swabs and TA were assessed using Pearson´s correlation coefficient.

## Results

Eighty-four healthy commercial Holstein-Friesian dairy calves aged 2–29 days were enrolled in the study. They originated from nine Danish dairy herds. The minimum and maximum number of calves from a herd was one and 27, respectively, with a mean of 9. The mean, median, minimum, and maximum concentrations of SAA, Hp and LBP in pharyngeal swabs, TA and serum are shown in Table 1.

**Table 1:** Concentrations of serum amyloid A (SAA), haptoglobin (Hp) and lipopolysaccharide binding protein (LBP) in serum, pharyngeal swabs, and tracheal aspirate fluid from 84 clinical healthy dairy calves, divided in age in days

	N	SAA mg/L	Hp mg/L	LBP mg/L
Overall serum	84	118.3 (131.7, 32.9, 180.9)	100.6 (77.6, 45.1, 788.8)	2.05 (1.90, 0.73, 5.63)
0–7 days	16	154.1 (153.5, 135, 180.9)	88.8 (78.5, 53.9, 230.3)	2.35 (2.47, 1.33, 3.30)
8–14 days	37	122.2 (134.5, 36.2, 179.8)	125.7 (77.6, 56.8, 788.8)	2.24 (1.93, 0.93, 5.63)
15–21 days	22	97 (84.5, 33.0, 178.0)	76.5 (77.2, 45.1, 99.7)	1.82 (1.63, 0.75, 4.41)
22–30 days	9	90.6 (87, 39.2, 164.4)	77.4 (77.6, 58.6, 87.5)	1.31 (1.10, 0.72, 2.25)
		SAA mg/L	Hp mg/L	LBP ng/mL
Overall pharyngeal swab	84	2.18 (1.97, 0.00, 8.01)	20.1 (18.1, 0.0, 60.8)	6.06 (0.00, 0.00, 47.94)
0–7 days	16	2.6 (2.3, 0.9, 6.7)	15.7 (12, 0, 58.6)	8.8 (0.0, 0.0, 47.9)
8–14 days	37	2.2 (1.9, 0, 8)	20 (16.6, 0, 55.9)	5.6 (0, 0, 33.4)
15–21 days	22	1.7 (1.2, 0, 4.4)	22.7 (22.6, 0, 43.1)	6 (0, 0, 42.6)
22–30 days	9	2.7 (2.9, 1.3, 4.7)	22.2 (19.9, 6.4, 60.8)	3 (0, 0, 21.4)
		SAA mg/L	Hp mg/L	LBP ng/mL
Overall tracheal aspirate	84	0.055 (0.042, 0.004, 0.240)	0.71 (0.39, 0.00, 5.89)	3.03 (2.72, 0.00, 13.22)
0–7 days	16	0.07 (0.053, 0.007, 0.143)	0.47 (0.29, 0.00, 2.15)	3.63 (3.12, 0.00, 13.22)
8–14 days	37	0.055 (0.04, 0.006, 0.24)	0.59 (0.37, 0.00, 2.55)	2.82 (2.61, 0.00, 11.52)
15–21 days	22	0.05 (0.043, 0.004, 0.164)	0.93 (0.71, 0.00, 3.05)	2.73 (1.47, 0.00, 8.99)
22–30 days	9	0.041 (0.042, 0.012, 0.07)	1.08 (0.43, 0.00, 5.89)	3.52 (3.49, 0.00, 8.78)

Mean (median, min, max), N = number of calves

**Fig. 1:**
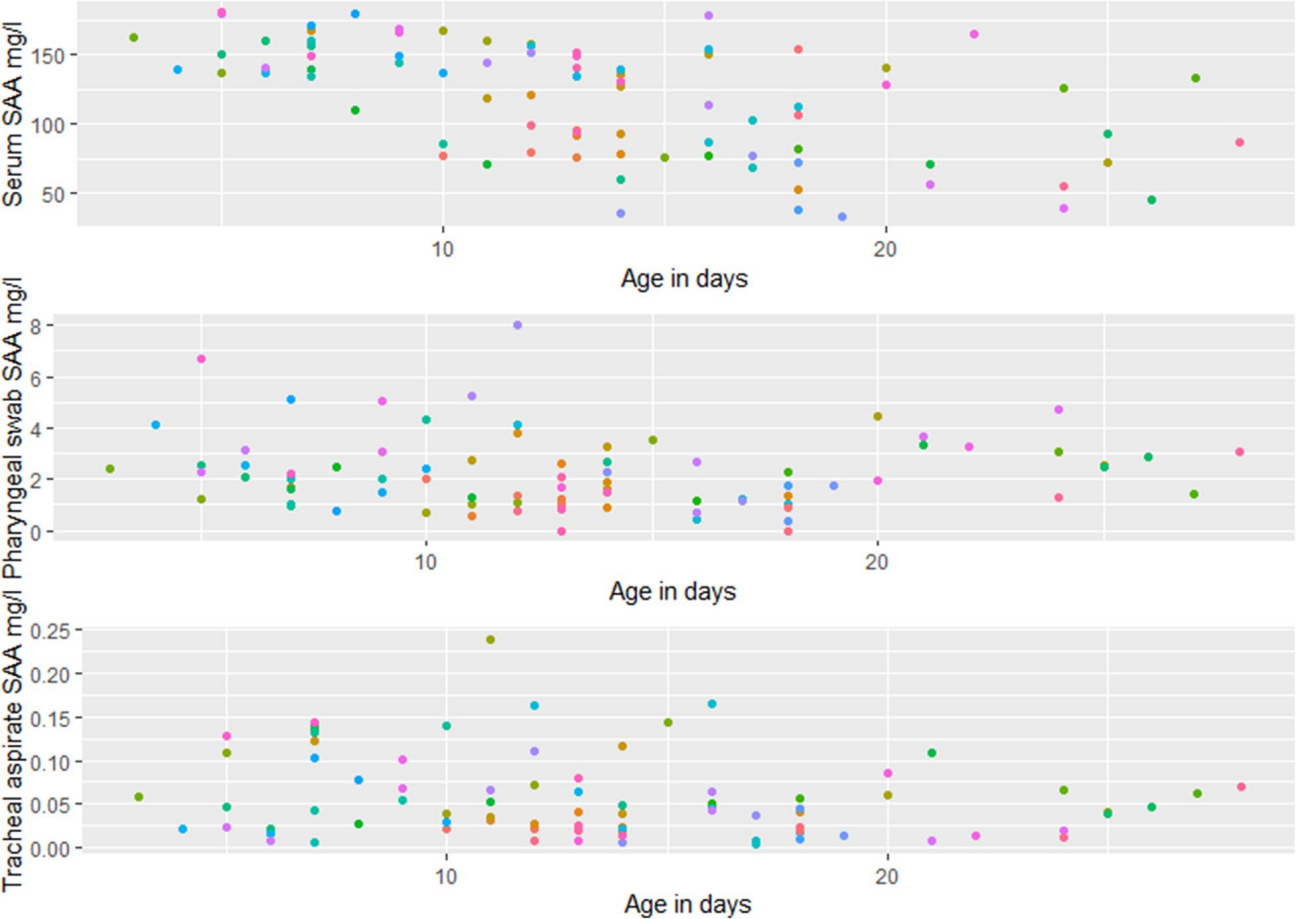
Serum amyloid A (SAA) concentrations as a function of age in days in serum, pharyngeal swabs, and tracheal aspirate fluid from 84 clinically healthy dairy calves. Samples from the same calf, are shown in the same colour

There was a statistically significant correlation (P < 0.05, r^2^ = 0.30) between serum SAA and age, with decreasing concentration with increasing age (Fig. 1—top). This age-related correlation was not found for SAA in pharyngeal swabs or TA. The concentration of SAA in serum ranged from 32.9 to 180.9 mg/L, in pharyngeal swabs from 0 to 8.01 mg/L and in TA from 0.004 to 0.240 mg/L. There were three calves with no detectable SAA concentration in the pharyngeal swab.

**Fig. 2:**
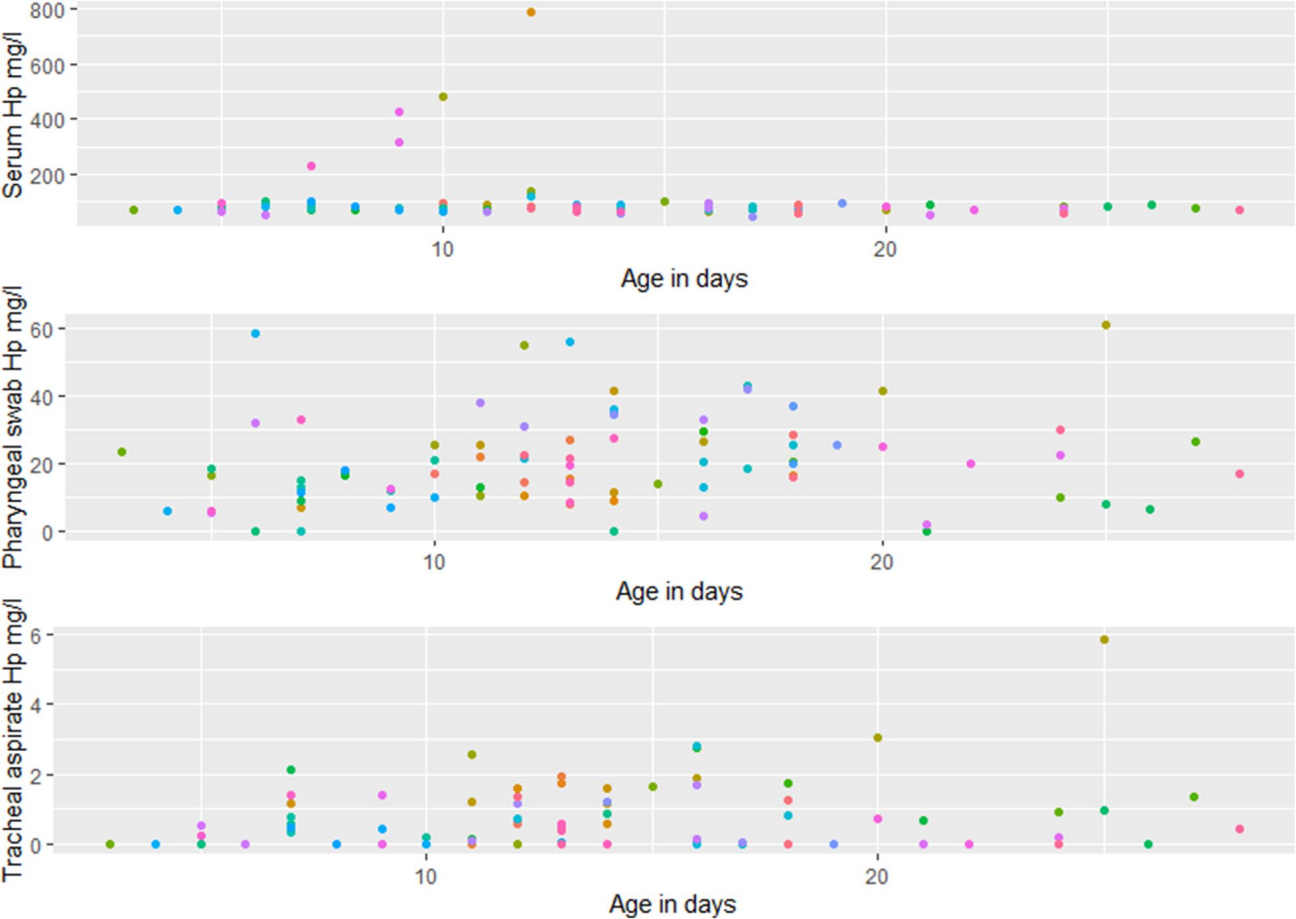
Haptoglobin (Hp) concentrations as a function of age in days in serum, pharyngeal swabs, and tracheal aspirate fluid from 84 clinically healthy dairy calves. Samples from the same calf, are shown in the same colour

The serum Hp concentration ranged from 45.1 to 788.8 mg/L, with all but five samples below 200 mg/L, which is the level found in healthy calves under 21 days of age [3]. The concentration in pharyngeal swabs varied from 0 to 60 mg/L, and the TA Hp concentration between 0 and 3 mg/L. Hp concentration was not statistically significantly associated with age in any of the fluids (Fig. 2). The concentration level of Hp was highest in serum, then pharyngeal swabs and least in TA. There were five calves with no detectable Hp concentration in pharyngeal swabs and 31 with no detectable Hp concentration in TA.

**Fig. 3:**
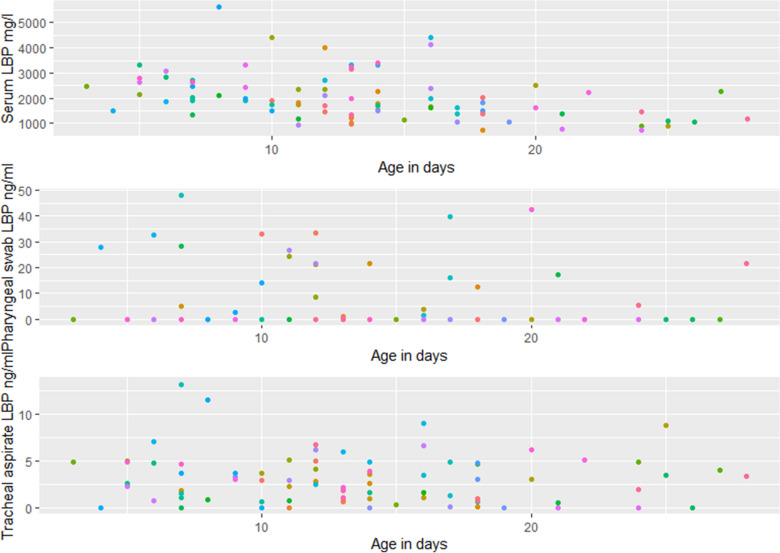
Lipopolysaccharide binding protein (LBP) concentrations as a function of age in days in serum and pharyngeal swab from 84 clinically healthy dairy calves. Samples from the same calf, are shown in the same colour

There was a statistically significant correlation (P < 0.05, r^2^ = 0.20) between serum LBP and age, with concentrations decreasing with age (Fig. 3 - top). This age-related correlation was not found for LBP in pharyngeal swabs or TA. The LBP concentration in serum ranged from 0.73 to 5.63 mg/L, in pharyngeal swabs from 0 to 47.94 ng/mL and in TA from 0 to 13.22 ng/mL. There were 59 and 9 calves with no detectable LBP concentration in the pharyngeal swab and TA, respectively.

**Fig. 4:**
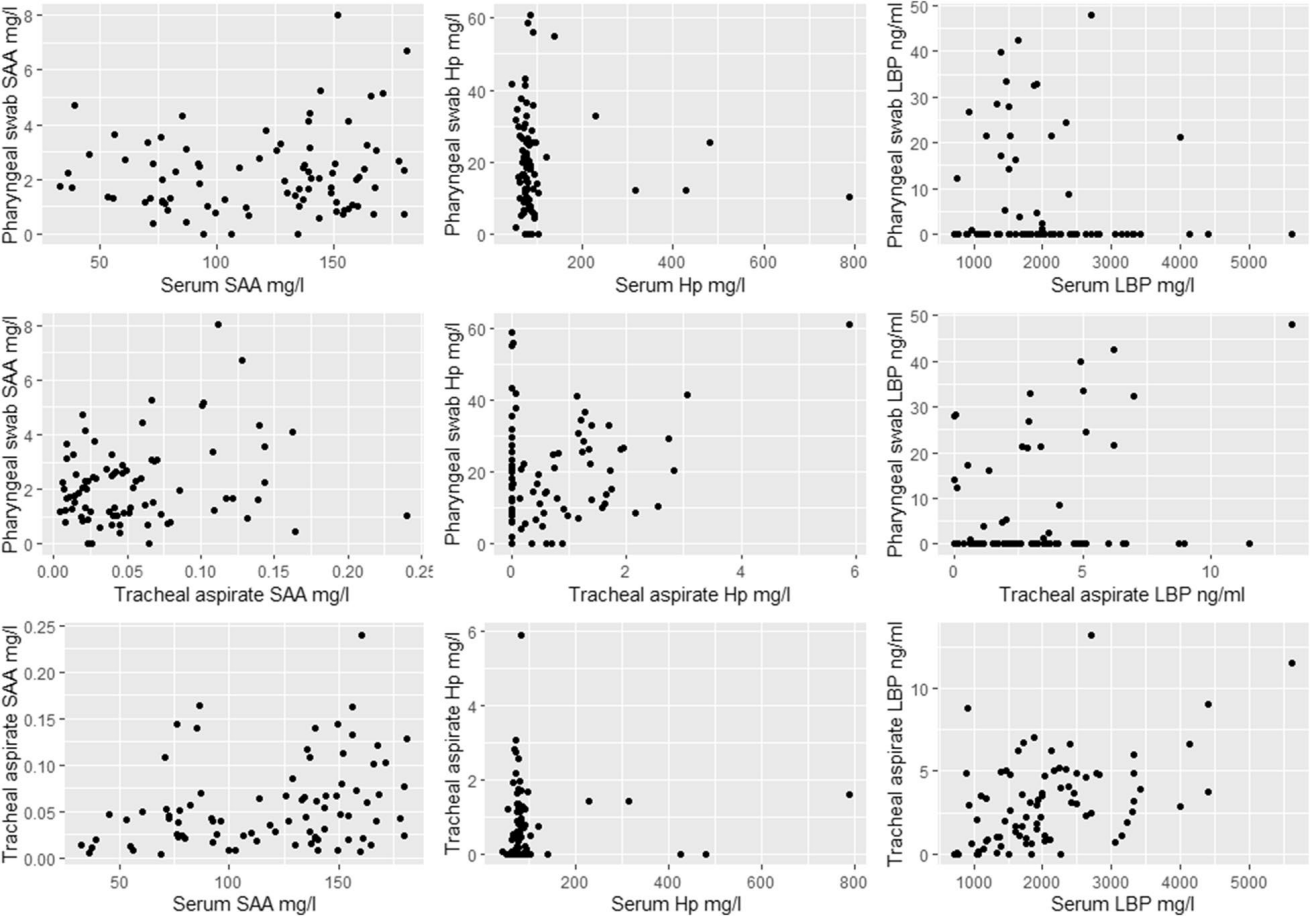
Correlations between Serum amyloid A (SAA), haptoglobin (Hp) and Lipopolysaccharide binding protein (LBP) in serum, pharyngeal swabs, and TA, from 84 healthy commercial dairy calves

The Pearson´s correlation coefficients between the concentration of SAA, Hp and LBP, in serum and pharyngeal swab and TA, and between TA and pharyngeal swabs ranged from − 0.15 to 0.49 (Table 2). The correlations are visualised in Fig. 4. The Pearson´s correlation coefficient between serum LBP and serum SAA was 0.65 and between serum SAA and serum Hp it was 0.19 (Table 2).

**Table 2:** Pearson´s correlation coefficients between the concentrations of Serum Amyloid A (SAA), haptoglobin (Hp) and lipopolysaccharide binding protein (LBP) in serum, pharyngeal swabs and tracheal aspirate (TA) from 84 heathy dairy calves

		Serum			TA		
		SAA mg/mL	Hp mg/mL	LBP mg/mL	SAA mg/mL	Hp mg/mL	LBP ng/mL
Pharyngeal swab	SAA mg/mL	0.13			0.2		
	Hp mg/mL		− 0.10			0.24	
	LBP ng/mL			− 0.15			0.26
TA	Hp mg/mL		0.05				
	LBP ng/mL			0.49			
Serum	Hp mg/mL	0.19					
	LBP mg/mL	0.65	0.39				

## Discussion

In this study we investigated the concentration of SAA, Hp and LBP in pharyngeal swabs, TA and serum from 84 healthy dairy calves. We found that these APPs could be detected in all the investigated body fluids, but in decreasing concentration in serum, pharyngeal swabs, and TA, respectively. We did not find a biologically interesting correlation or relationship between the concentration of SAA in serum and either pharyngeal swab or TA, nor between TA and pharyngeal swab. The same applied for Hp and LBP.

### Pharyngeal swabs

We explored for the first time the concentrations of SAA, Hp and LBP in pharyngeal swabs from healthy calves. For all APPs, there were samples with APP concentration below the detection limit. In particularly LBP, where 59 of 84 samples had a concentration below the detection limit. For SAA and Hp, it was only 3 and 5, respectively. Saliva is a liquid containing more than 99% water [18], which is then diluted more by placing the cotton swabs in PBS. With this in mind it is not surprising that the APP concentrations were low. This was also the rationale for not diluting the samples further during analysis. When collecting the pharyngeal swabs, we noticed that the amount and viscosity of pharyngeal saliva varied a lot between calves, which is likely to have caused the amount of saliva in the samples to vary. Even though we corrected the APP concentration for the dilution effect of the PBS, the explanation for some of the variance in APP concentration, could be a technical difference introduced by the sampling method. The number of pharyngeal swab samples with very low concentrations of LBP were too high to be explained by technical issues alone. In particular, since there were detectable concentrations of SAA and Hp in nearly all the samples with very low concentrations of LBP. There might be very low concentrations of LBP in pharyngeal swabs in healthy calves, but if this increases significantly with inflammation, LBP in saliva might still be a potential indicator of airway inflammation. However, LBP in saliva needs to be explored further to conclude on its potential as a biomarker.

The pharyngeal swabs were collected under light sedation. This was done because we also collected TA samples. However, the collection of pharyngeal swabs can also be done without sedation, as a non-invasive procedure. Caplen and Held [14] collected saliva from cows using a cotton swab developed for saliva collection in children without sedation of the cows, which would presumably also work in calves.

Rahman et al. [15] have reported measuring Hp concentrations in saliva from calves before dehorning. Unfortunately, they do not elaborate further on the method of collection and whether or not the calves were sedated for the procedure.

The calves enrolled in our study were young and still milk-fed. This could be a potential bias, as there could be traces of APP from milk in their pharynx. This is unlikely to have impacted the results, since all samples were sampled at least 2 h after milk feeding.

### TA

The SAA concentration in TA ranged from 0.004 to 0.240 mg/L, with an average of 0.055 mg/L, Hp ranged from 0.00 to 5.89 mg/L, with an average of 0.71 mg/L and LBP ranged from 0 to 13.22 ng/mL, with an average of 3.03 ng/mL. Prohl et al. [10] found a median Hp concentration in TA in healthy calves of 0.1073 mg/L, ranging from 0.003 to 0.347 mg/L and Coskun et al. [11] found a mean of 0.03 mg/L and standard deviation of 0.007 mg/L. The median LBP concentration in healthy calves were 9.5 ng/mL, ranging from 1.7 to 23.6 ng/mL [10]. We believe that our TA results are largely in line with findings by the authors mentioned above, because there is an unknown dilution factor of the TA samples. Even though all calves were flushed with the same amount of NaCl, there were differences in how much fluid it was possible to aspirate, and we were not able to take account of this variation because the exact concentration of the TA was unknown to us.

Based on this study, we are not able to determine how the APP concentrations in pharyngeal swabs change during respiratory disease. SAA, Hp and LBP in TA increases in calves with respiratory disease [10, 11], but a validation of the used assays in this material have not been performed. Rahman et al. found that saliva Hp increased mildly 24 h after dehorning [15], indicating that there is a potential for APP in saliva to differentiate between healthy and diseased calves. As mentioned, for both pharyngeal swabs and TA there is a considerable technical variation in the sampling methods, but if the true difference in APPs between healthy and diseased calves is large enough, this would overcome the technical variation and still be promising for detecting respiratory disease in calves.

For our analysis of the APP concentration in pharyngeal swabs and TA, we used ELISA kits not validated in these materials. Full interpretation of the data and calculation of reference intervals for APPs in TA and pharyngeal swabs requires a validation of the assays using the biological materials TA and saliva.

### Serum

The serum SAA concentrations of the calves in this study are supported by the results of other studies of serum SAA from healthy calves, where the concentrations range from 80 to 200 mg/L [2, 4]. As also found in other studies [3, 4], the serum SAA concentrations of the calves in this study were associated with age, with a higher concentration in young calves. The few calves with high serum Hp concentrations are discussed in detail in a later section.

The concentration of serum LBP ranged from 0.72 to 5.63 mg/L, with an average of 2.05 mg/L. Serum LBP in a group of slightly older healthy calves was found to be 1.5-3 mg/L [8] and 1-69.8 mg/L [10]. This seems to be close to our samples, even though Prohl et al. found a wider range than our samples [10]. However, as the same test kits have not been used, there can be differences between laboratories and differences in sample size. Orro et al. [3] also investigated serum LBP in calves less than 21 days of age using the same ELISA kit, as used in our study. They found a higher mean serum LBP concentration ranging from 16 to 32 mg/L and with a decreasing serum LBP with increasing age up to approximately 20 days of age. Based on the results of our study and other published studies, there is a considerable variation in serum LBP concentration in healthy calves.

### Correlations

Based on the Pearson´s correlation coefficients being close to zero for most correlations between the concentration of the same APP in serum and TA or pharyngeal swab, we find there were no biologically interesting correlations between the APP concentrations in the different fluids. A linear correlation between serum and pharyngeal swabs or TA, would indicate a direct reflection of the specific APP concentration in serum in the bronchial fluid and the mucous membranes of the pharynx. Rahman et al., found a moderate correlation between serum and saliva SAA in calves 24 h after dehorning [15], but this is to our best knowledge the only previous investigation. Coskun et al. and Prohl et al. investigated concentrations of SAA, Hp and LBP in paired serum and TA samples, but none of the authors present the results for individual calves [10, 11]. Therefore, it is not possible to determine if they found a correlation in the APPs between the different fluids.

The correlations of APP concentrations between serum and TA or pharyngeal swab could also be time-shifted, e.g., first there is a rise in serum, which is followed in pharyngeal swabs or TA after some time. Such a correlation would require repeated measurements on the same calves, and as we have cross-sectional data, this cannot be investigated in this study.

Another explanation could be that the APP concentration in pharyngeal swab or TA, only rises in case of respiratory disease. Others have shown that the concentration of SAA, Hp and LBP in TA increases with respiratory disease [10, 11] and salivary Hp increases after dehorning [15]. We might have seen a detectable association if we had investigated diseased calves.

### Clinical status

Although only clinically healthy calves were enrolled in the study, concentrations of serum Hp was above 200 mg/L in five calves. Hp has a longer half-life in serum than other APPs [9] and the Hp concentration can possibly be above the cut-off in a clinically healthy calf, because of previous disease, not evident anymore. All but one of the five calves with a higher serum Hp concentration, were observed with mild diarrhoea and no signs of dehydration, which could explain the high Hp concentration [19, 20]. Other authors have had the same experiences and highlight the differences in getting a homogenous sample of calves in commercial herds for evaluation of APP concentrations [3].

## Conclusions

This study showed that concentrations of the APPs SAA, Hp and LBP can be measured in pharyngeal swabs and TA from healthy dairy calves. We did not find biologically interesting correlations between the specific APP concentration in serum and pharyngeal TA nor swab. A considerable technical variation in the chosen sampling method for both pharyngeal swabs and TA exist, and further validation of the methods are needed. However, if the APP concentration in saliva is elevated in diseased calves, there is a great potential for using pharyngeal swabs as a less invasive method for detection and monitoring of disease in calves.

## Data Availability

The datasets used and analyzed during the current study are available from the corresponding author on reasonable request.

## Abbreviations

APP: Acute phase protein
BALF: Bronchoalveolar fluid
Hp: Haptoglobin
LBP: Lipopolysaccharide binding protein
SAA: Serum amyloid A
TA: Tracheal aspirate
